# Strong Positive Correlation between TSH and Ghrelin in Euthyroid Non-Growth Hormone-Deficient Children with Short Stature

**DOI:** 10.3390/molecules25173912

**Published:** 2020-08-27

**Authors:** Katarzyna Adamczewska, Zbigniew Adamczewski, Anna Łupińska, Andrzej Lewiński, Renata Stawerska

**Affiliations:** 1 Department of Endocrinology and Metabolic Diseases, Polish Mother’s Memorial Hospital—Research Institute, 93-338 Lodz, Poland; kadamczewska@o2.pl (K.A.); zbigniew.adamczewski@umed.lodz.pl (Z.A.); ankalupinska@op.pl (A.Ł.); andrzej.lewinski@umed.lodz.pl (A.L.); 2 Department of Endocrinology and Metabolic Diseases, Medical University of Lodz, 93-338 Lodz, Poland; 3 Department of Pediatric Endocrinology, Medical University of Lodz, 93-338 Lodz, Poland

**Keywords:** ghrelin, growth hormone, thyroid stimulating hormone, free thyroxine, free triiodothyronine, insulin-like growth factor I, idiopathic short stature, children

## Abstract

The growth processes in children depend on the proper functioning of some hormones and growth factors. Recently, a positive correlation between ghrelin and TSH (thyroid stimulating hormone) in patients with hyper- and hypothyroidism was proved. Moreover, in hypothyroid rats with high ghrelin concentration, growth hormone (GH) and insulin-like growth factor I (IGF-I) secretion was suppressed. We analyzed these relationships in euthyroid prepubertal children with idiopathic short stature (ISS). The analysis comprised concentration of ghrelin, GH in stimulating tests and during the night, as well as IGF-I, TSH, free thyroxine (FT4) and free triiodothyronine (FT3) in 85 children with ISS (36 girls, 49 boys) aged 9.65 ± 3.02 years (mean ± SD). A strong positive correlation between ghrelin and TSH was confirmed (r = +0.44, *p* < 0.05). A higher ghrelin but lower nocturnal GH and lower IGF-I were observed in children with higher normal TSH concentration than those in children with lower normal TSH. Interestingly, alterations of TSH level were without any impact on FT4 and FT3 concentrations. Summing up, in ISS prepubertal euthyroid children, ghrelin and TSH secretion are closely related. On the other hand, the higher the TSH, the lower the nocturnal GH and IGF-I levels. The contribution of the above findings in deterioration of growth processes requires further studies.

## 1. Introduction

Human postnatal growth processes are determined by the proper action of two axes: growth hormone (GH)—insulin-like growth factor I (IGF-I) and thyroid stimulating hormone (TSH)—thyroid hormones (thyroxine—T4 and triiodothyronine—T3). GH regulates cell growth, differentiation, apoptosis, and reorganization of the cytoskeleton; these effects are mediated through IGF-I, which is produced mainly in the liver in response to GH [1]. GH secretion is stimulated directly by GH releasing hormone (GHRH), as well as by ghrelin; the latter hormone additionally can exert an indirect effect [2]. It is worth mentioning that ghrelin is also an orexigenic hormone involved in energy balance regulation [3,4]. In turn, thyroid hormones regulate growth of long bones, protein synthesis, as well as the neuronal proliferation, migration and maturation; they also increase the basal metabolic rate (BMR).

Mutual relationships between growth-related molecules of the above mentioned axes are observed. Free T4 (FT4) and free T3 (FT3) exert a permissive impact on IGF-I action; it was demonstrated that hypothyroidism, even in its subclinical form, affected IGF-I secretion [5]. Moreover, in short children with FT4 levels within the lowest third of normal range, administration of L-thyroxine (L-T4) improved growth rate and IGF-I response to GH [6]. On the other hand, GH influences the monodeiodination of FT4 to FT3; this reaction is also mediated by IGF-I [7]. In many children with GH deficiency, shortly after the start of GH replacement therapy, FT4 levels significantly decreased [8,9].

Therefore, in the diagnostic process of children with short stature, the presence of GH deficiency, primary IGF-I deficiency, hypothyroidism, as well as the chronic disorders which cause the secondary IGF-I deficiency, should be taken into consideration [10,11]. After these disorders (as well as other known causes of short stature, e.g., genetic disorders) are excluded, an idiopathic short stature (ISS) can be suspected [10] and further watchful waiting is recommended. Unfortunately, some of these children do not achieve predicted final height.

It was reported that in about 40% of children with ISS, a reduced IGF-I concentration was observed, due to unknown causes [12]. Thus, the pathomechanism of the growth processes is still not well explained. In our previous studies, we also noted that among children with ISS, there were a lot of cases with reduced IGF-I concentration [13]. Moreover, the lower the IGF-I was observed, the higher ghrelin concentration was confirmed in this group of children. In ISS group we also found the tendency for ghrelin concentration being higher than in controls [14].

On the other hand, the correlations between ghrelin and TSH or FT4 levels in patients with hypo- and hyperthyroidism have been studied in numerous analyses. It has been documented that ghrelin level is elevated in severe hypothyroidism and reduced in hyperthyroidism, when compared to controls [15]. Thus, in such cases, ghrelin concentration is negatively correlated with FT4 and FT3 and positively with TSH. It has also been shown that ghrelin levels increase when patients with hyperthyroidism develop hypothyroidism after ^131^I radioiodine therapy [16,17].

Chang et al. observed that in hypothyroid rats with confirmed high ghrelin concentration, GH level was not elevated, while IGF-I secretion was even suppressed [18]. However, it should be stressed that studies concerning the relationships among hormones that are crucial for the growth processes in a group of ISS children have so far been scarce.

Thus, the aim of the present study has been to analyze the mutual relations among ghrelin, GH, IGF-I, TSH, FT4 and FT3 in ISS euthyroid children (non-GH-deficient); three (3) problems to be elucidated have arisen: (1) whether elevated levels of ghrelin (observed in some ISS children) have any effect on TSH or FT4 and FT3 secretion, the phenomenon which may be responsible for their slow growth velocity; (2) whether the ISS children with FT4 concentration in the lower normal range are characterized by reduced IGF-I and/or by increased ghrelin concentration; and (3) should L-T4 treatment be recommended for them.

## 2. Results

**Figure 1:**
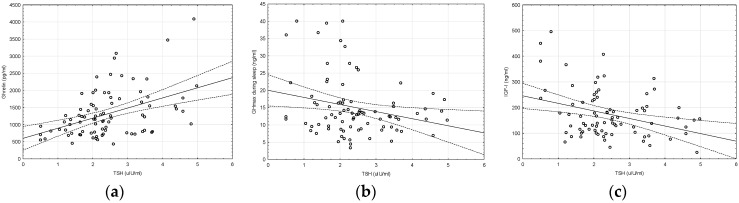
Correlation between: (**a**) TSH (thyroid stimulating hormone) and ghrelin concentrations (r = +0.44, *p* < 0.05); (**b**) TSH concentration and GHmax concentration during sleep (r = −0.25, *p* < 0.05); (**c**) TSH and IGF-I concentrations (r = −0.3, *p* < 0.05).

In the analyzed group of prepubertal euthyroid children with ISS, a significant positive correlation between ghrelin and TSH concentration was confirmed; however, a correlation between ghrelin and FT4 (or FT3) concentration was not noticed. Moreover, a significant negative correlation between TSH and maximal GH concentration during sleep and between TSH and IGF-I concentration was observed (Figure 1).

**Figure 2:**
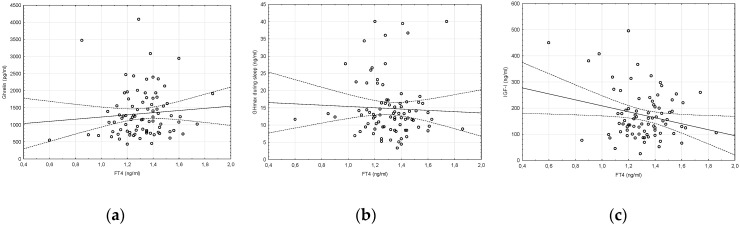
Correlation between: (**a**) FT4 and ghrelin concentrations (r = +0.08, *p* > 0.05); (**b**) FT4 and GHmax during sleep (r = −0.04, *p* > 0.05); (**c**) FT4 and IGF-I concentrations (r = −0.19, *p* > 0.05).

Correlations between TSH and maximal GH levels in individual stimulation tests, or between TSH and FT4 (or FT3) levels, were not observed. On the other hand, we did not find any significant correlations between FT4 levels and ghrelin concentration, maximal GH concentration during sleep or IGF-I concentration (Figure 2).

**Figure 3:**
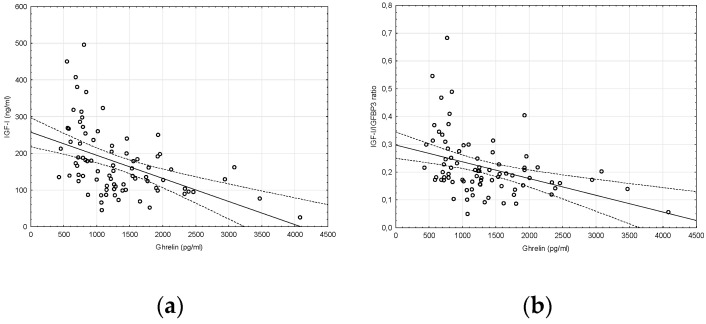
Correlation between: (**a**) ghrelin and IGF-I concentrations (r = −0.47, *p* < 0.05); (**b**) ghrelin concentration and IGF-I/IGFBP-3 molar ratio (r = −0.27, *p* < 0.05).

In turn, the significant negative correlation between ghrelin and IGF-I levels as well as between ghrelin levels and IGF-I/IGFBP-3 (insulin-like growth factor binding protein-3) molar ratio were noticed (Figure 3).

Taking into consideration the positive correlation between ghrelin and TSH levels, we decided to analyze our group of children after dividing them into two subgroups based on TSH median value: children with TSH value below 2.29 µIU/mL (lower normal TSH) and children with TSH value ≥ 2.29 µIU/mL (higher normal TSH). Higher ghrelin concentration but lower IGF-I and GH levels during the night were confirmed in children with TSH concentration equal to or above 2.29 µIU/mL when compared to children with TSH below 2.29 µIU/mL. Interestingly, there were no differences in FT4 and FT3 concentrations between the subgroups (Table 1).

A multivariate analysis shows that age, sex and body mass have no impact on this relationship. A higher fasting insulin and IRI HOMA in the group of children with lower normal TSH vs. upper normal TSH was noticed, while glucose and insulin concentration at the 120th minute of OGTT was significantly higher in children with higher vs. lower TSH. No differences were observed between the groups regarding lipids, leptin, resistin or adiponectin.

Next, based on FT4 value, we divided the analyzed group of children into two subgroups: children with FT4 results remaining in the lowest tercile of the reference range and children with FT4 concentration equal to or being above the upper limit of lowest tercile (i.e., remaining in the middle or highest tercile of the reference range). There were no significant differences in the auxologic parameters (growth deficiency or BMI), ghrelin, GH, IGF-I and/or TSH concentration. No differences between groups in regards to glucose, insulin, lipids, leptin, resistin, adiponectin were observed, either. The significant differences in FT4 and FT3 concentration result from the essence of the division used (Table 2).

Similarly, when we divided our group into 2 subgroups according to FT4 median value (1.3 ng/mL; the first group below the median value, the second equal to or above 1.3 ng/mL), no differences for any of the analyzed parameters were found.

## 3. Discussion

In our present study, we have attempted to clarify the relationships between two growth regulatory feedback loops: ghrelin-GH-IGF-I and TSH-FT4/FT3 in euthyroid short children diagnosed with ISS. The initial point for us was the observation that in about 40% of children in whom “idiopathic” short stature was diagnosed, low IGF-I concentrations were observed [12]. In our earlier study, we also confirmed these findings [13]. In our research, we considered various causes of low IGF-I (secondary IGF-I deficiency), one of them being the reduction in IGF-I production as a result of chronic disorders, mainly gastrointestinal tract diseases or malnutrition (due to the mechanism of sirtuins dysfunction). For this reason, the exclusion criteria included malnutrition and a history of chronic diseases, as well as abnormalities found during physical examination. Therefore, we believe that the cause may be a disturbance of the GH secretagogue type 1a receptor or GH receptor activity or others. Moreover, we also showed a negative correlation between IGF-I and ghrelin concentration [19]. As ghrelin is a stimulating factor for GH secretion, and, in turn, GH stimulates IGF-I secretion in healthy children, we have concluded that in short children a low concentration of IGF-I triggers feedback mechanisms, i.e., higher production of ghrelin. The causes of low IGF-I secretion observed in these children are not fully understood and other mechanisms responsible for this phenomenon should be sought.

In most human studies based on the results obtained from patients with hyperthyroidism and hypothyroidism, a positive correlation between ghrelin and TSH was confirmed [15,16,17,20]. In our present study, we have also confirmed a strong positive correlation between TSH and ghrelin concentration in the group of children that we analyzed. However, it should be emphasized that our study included only euthyroid children. The authors cited above have suggested that the fluctuation of ghrelin secretion acts as a compensatory factor, helping to balance metabolic disturbances in patients with hyperthyroidism and hypothyroidism. However, it is to be recalled that short children analyzed by us, were euthyroid. Therefore, the explanation for these findings seems to be different.

It has been proved that ghrelin is able to stimulate TSH from thyrotropic cells of anterior pituitary and the density of ghrelin receptors seems to increase in mice when the food intake is not sufficient [21]. In our previous study, we noticed that ghrelin secretion was higher in slim children, compared to children with normal body weight [19]. It is well known that ghrelin, apart from being a GH stimulatory factor, is also an orexigenic hormone that regulates appetite and affects metabolic homeostasis [3,4]. Thus, in contrast to GH, ghrelin secretion is expressed not only during the night but also during the day and its production depends on food intake: it increases in the fasting state and decreases after meals [22,23]. Moreover, ghrelin levels negatively correlate with body mass: its concentration increases in malnutrition and decreases in obesity [24,25]. Although a negative correlation between ghrelin concentration and BMI SDS was found in the analyzed group of ISS children, when we divided them into two subgroups (one with low normal TSH and one with high normal TSH) we did not observe any differences as regards the nutritional status of the children, expressed by BMI SDS. Therefore, the nutritional status of the children was not a significant factor in the issue under consideration. It was also confirmed by the results of the multivariate analysis.

Since we observed a positive correlation between ghrelin and TSH concentration, we assumed that increased ghrelin level (which is probably a response to low IGF-I formation) stimulated the release of TSH from the pituitary gland. Thus, it was surprising that we did not observe any effect on FT4 levels. Ghrelin receptors were also found on human thyrocytes; the indirect suppression effect of ghrelin on thyrocytes was documented by Barington et al. in 2017 [26]. The authors have concluded that ghrelin possesses the ability to reduce TSH-induced thyroglobulin level through the deterioration of thyroid peroxidase (TPO) expression. Therefore, we speculate that this may be a reason why FT4 concentration is not elevated, despite higher TSH concentration. Furthermore, no correlation between ghrelin and FT4 secretion, or between FT4 and IGF-I secretion, was found. It is an important issue for further consideration. Hypothyroidism, even in its subclinical form, is known to affect IGF-I [5] secretion and the administration of L-thyroxine (L-T4) improves growth rate and IGF-I response to GH in short children with lower normal FT4 levels [6]. Thus, based on the results of our analysis, we did not find objective evidence that the application of L-T4 treatment in children with low normal FT4 effectively improved their growth rate, just as we did not observe any correlation between FT4 and IGF-I.

In our study we also analyzed the FT4/FT3 ratio. Although the effect of IGF-I on deiodination of FT4 to FT3 is well known, in the group of children we have analyzed, both FT4 and FT3 levels have not differed between the group with higher ghrelin and lower IGF-I and the group with lower ghrelin and higher IGF-I. About 80% of the deiodination takes place in peripheral organs, such as the liver and kidney, and occurs intracellularly, while the concentrations measured in the blood represent the level of FT4 and FT3 secretion from the thyroid gland, which is about 20% of deiodination. We believe that this is the reason why the FT4/FT3 ratio does not differ between groups.

It is interesting that the higher the TSH and ghrelin are, the lower the GH concentration is during the night and the lower the IGF-I. Boulenger et al. [27] have proved that in hypothyroid rats, GHRH receptors in the anterior pituitary gland are down-regulated, which may result in a reduced GH production after GHRH stimulation. A similar relationship was also observed by Chang et al. [18]. They analyzed the results of TSH, ghrelin, GH and IGF-I in rats, divided into four groups, according to four (4) procedures performed: thyroidectomy (Tx), sham Tx, Tx + L-T4 therapy and after propylothiouracyl (PTU) injection. The authors observed that in rats, both after Tx and PTU (i.e., with primary hypothyroidism and with higher TSH), ghrelin increased by 75% and GH secretagogues receptor type 1 (GHS-R1) expression in the anterior pituitary was up-regulated. However, in both groups with hypothyroidism, similarly to the results of our study, it did not cause a significant increase in GH secretion, while IGF-I concentration decreased by 51% and 63%, respectively.

In our study, as in the report by Chang et al. [18], we also observed differences between IGF-I concentration in individual groups; IGF-I was negatively correlated with TSH and ghrelin, positively with nocturnal GH. Surprisingly, this correlation was not observed for the GH results obtained during GH stimulation tests, routinely used in GHD diagnostics.

Chang et al. [18] concluded that hypothyroidism disrupts ghrelin/GHS-R axis for stimulating GH secretion. The relationships presented by authors in the mentioned study [18] concerned cases with hypothyroidism, while the group of children we analyzed did not have a thyroid gland dysfunction. However, some analogies arise. It seems that it is worth discussing the normal range for TSH concentration in children with ISS, since after L-T4 administration in young adult rats, in Chang et al.’s study [18], ghrelin concentration decreased, GH secretion was enhanced and IGF-I concentration normalized.

Summing up, we observed a positive correlation between ghrelin and TSH in euthyroid children with idiopathic (non-GH deficit) short children. It was confirmed that the higher the ghrelin and TSH, the lower the GH secretion during the night and the lower the IGF-I. However this is without any impact on FT4 concentrations.

We speculate that in children with ISS in whom ghrelin concentration is elevated (probably in response to low IGF-I secretion), TSH impact on FT4 secretion from thyrocytes is reduced by higher ghrelin concentration. In the feedback mechanism, increasing secretion of TSH from the pituitary gland for the purpose of FT4 normalization is observed.

The second hypothesis is that due to higher ghrelin concentration, the stimulation of TSH secretion from pituitary gland is enhanced. However, due to a weaker impact of TSH on thyrocytes under this condition (high ghrelin concentration), the FT4 concentration is not elevated and stays at the same level as in children with lower TSH.

It is possible that relative hypothyroidism disrupts the ghrelin/GHS-R axis impact on stimulating GH secretion, as GH secretion at night is disturbed in these children (while GH results obtained during GH stimulation tests, routinely used in GHD diagnostics, are normal). Further studies are needed to explain these findings.

Limitation of the study: Certainly, employing a control group consisting of healthy children with normal height would have facilitated the interpretation of the results. In addition, generally accepted reference values for ghrelin concentration are not yet available. Stimulation tests for GH secretion are still the basic tool in the diagnosis of GHD, however when drawing conclusions from the test results, it must be taken into account that their reproducibility creates some problems.

## 4. Materials and Methods

The study was approved by the Bioethical Committee at the Polish Mother’s Memorial Hospital - Research Institute (PMMH-RI) in Lodz.

The children were recruited over a period of 18 months from patients of the Outpatient Clinic of PMMH-RI, where they had been referred due to short stature. In all the children, height and body mass were measured, using a stadiometer and scales, respectively. Next, the height standard deviation score (height SDS) and body mass index standard deviation score (BMI SDS) were calculated, based on the current population standards data, given by Palczewska and Niedźwiecka [28]. The stage of puberty was assessed according to the Tanner’s scale [29]. Next, a detailed medical history was collected and the thyroid function was assessed in each child, based on TSH and FT4 serum concentrations. All children qualified for the study lived in areas of sufficient iodine supply.

Exclusion criteria: (1) –height SDS above −2.0 SD, (2) undernutrition and obesity (BMI SDS above +2.0 SD or below −2.0 SD for the reference range for age and sex [28]), (3) puberty stage more than Tanner I stage, (4) history of chronic diseases, dysmorphic features or abnormalities found during the physical examination, (5) abnormal results of TSH and/or FT4.

Next, the children were diagnosed at the Department of Endocrinology and Metabolic Diseases of PMMH-RI, to assess GH secretion and IGF-I concentrations.

In each child, a 3-h, nocturnal profile of GH secretion was recorded every half-hour, starting from the first hour after falling asleep.

Then, two GH-stimulation tests were performed on subsequent days of hospitalization. In the first of them, GH was measured before (0 point) and at the 30th, 60th, 90th and 120th minute after oral clonidine administration in dose of 0.15 mg/m^2^ of body surface. In the second one, GH was measured before (0 point) and at the 90th, 120th, 150th and 180th minute after intramuscular glucagon administration in dose of 30 µg/kg (not exceeding 1 mg). Peak GH concentration (GH_max_) was determined in both tests and after falling asleep. The cut-off value for normal and subnormal GH peak in response to stimulation was 10.0 ng/mL, according to current recommendations [30,31].

In children with GH_max_ values < 10 ng/mL, GHD was recognized and those cases were excluded from further analysis, while in children with normal value of GH_max_ (more than 10 ng/mL), ISS was diagnosed and those cases were qualified into the study group.

Finally, 85 children with ISS (36 girls and 49 boys), aged 9.65 ± 3.02 years (mean ± SD), from 4.6 to 12.4 years were selected for the study group.

In each child, in the morning, in fasting state, ghrelin, IGF-I, IGFBP-3, TSH, FT4, FT3, anti-tyreoperoxidase (aTPO) and anti-tyreoglobulin (aTg) antibodies, lipids, glucose, insulin, leptin, adiponectin and resistin concentrations were measured in single blood samples. Additionally, in 28 children, oral glucose tolerance tests (OGTT) were performed including an assessment of glucose and insulin at 0′, 60′ and 120′ minutes from oral glucose administration (dose 1.75 g/kg body mass, max. 75 g).

The concentrations of GH were measured by a two-site chemiluminescent enzyme immunometric assay (hGH IMMULITE, DPC) for the quantitative measurement of human GH, calibrated to WHO IRP 80/505 standard. The analytical sensitivity of the assay was up to 0.01 ng/mL, the calibration range up to 40 ng/mL, the sensitivity of 0.01 ng/mL, the intra-assay coefficient of variation (CV) of 5.3–6.5% and the inter-assay CV of 5.5–6.2%.

The total ghrelin concentration was measured using the Millipore RIA kit (Linco Research, St. Charles, MO, USA) with sensitivity level: 100–10.000 pg/mL, the intra-assay CV: 3.3–10.0% and inter-assay CV: 14.7–17.8%.

Both IGF-I and IGFBP-3 concentrations were assessed with Immulite, DPC assays. For IGF-I, WHO NIBSC 1st IRR 87/518 standard was applied, with the analytical sensitivity of 20 ng/mL, calibration range up to 1600 ng/mL, intra-assay CV: 3.1–4.3% and inter-assay CV: 5.8–8.4%. The assay for IGFBP-3 assessment was calibrated to WHO NIBSC Reagent 93/560 standard, with analytical sensitivity 0.02 μg/mL, the calibration range up to 426 μg/mL, the intra-assay CV: 3.5–5.6% and the total CV: 7.5–9.9%.

For the calculation of IGF-I/IGFBP-3 molar ratio, the following molecular masses were used: 7.5 kDa for IGF-I and 42.0 kDa for IGFBP-3 [32,33].

Serum TSH, FT4 and FT3 concentrations were measured by the electroimmuno-chemiluminescent method (ECLIA), Roche, Elecsys ^®^ Systems 1010/2010/modular analytics E170. For TSH, the analytical sensitivity was 0.005 μIU/mL, range up to 100 μIU/mL, intra-assay coefficient of variance (CV) 1.5–8.6%, accuracy 1.1–3.0%. The analytical range for FT4 was 0.023–7.77 ng/mL, intra-assay CV 1.4–2.9%, accuracy 2.7–6.6%. For FT3, the analytical range was 0.26–32.55 pg intra-assay CV 3.7–9.5%, accuracy 3.8–11.2%. For the assessment of FT3/FT4 molar ratio, the concentrations of FT4 and FT3 were expressed as molar ones.

The leptin, resistin and adiponectin concentrations were measured using the Millipore ELISA kit (Linco Research). The sensitivity level, the intra-assay CV and inter-assay CV were: 0.5–100 ng/mL, 1.4–4.9% and 1.3–8.6% for leptin; from 0.16 ng 3.2–7.0% and 7.1–7.7% for resistin and from 0.78 ng/mL, 7.4% and 2.4–8.4% for adiponectin, respectively. Based on fasting adiponectin and resistin concentrations, the adiponectin-resistin (AR) index, according to the formula proposed by Lau and Muniandy [34], was calculated: 1+log10 (fasting resistin)-log10 (fasting adiponectin).

Plasma insulin concentration was measured using the DRG ELISA kit; sensitivity level 1.76 – 100 µIU/mL, the intra-assay CV: 1.8–2.6 and inter-assay CV: 2.9–6.0. Plasma glucose concentration was determined using the enzymatic method, with the use of hexokinase.

Based on the results of fasting glucose and insulin concentration, insulin resistance index was calculated [35], according to the homeostasis model assessment (IRI HOMA): fasting glucose [mmol/L] x fasting insulin [µIU/mL])/22.5.

Based on the glucose and insulin concentration during OGTT, IRI_Belfiore_ was calculated according to the formula [36] = 2/{[1/(GLU_AUC_ x INS_AUC_)]+1}, where: GLU_AUC_ = GLU_AUCi_/GLU_AUCmean,_ while INS_AUC_ = INS_AUCi_/INS_AUCmean_, where GLU_AUCi_ and INS_AUCi_—areas under respective glucose or insulin concentration curve during OGTT in a given patient, while GLU_AUCmean_ and INS_AUCmean_—areas under respective glucose or insulin concentrations during OGTT for an age group for our population (those values were calculated in our earlier study) [37].

The data were analyzed using Statistica 11.0 software (StatSoft, Inc., Tulsa, OK, USA). The continuous variables were expressed as mean ± standard deviation for normally distributed variables. Shapiro–Wilk’s test was used to test the distribution of the variables. The differences between the sexes were compared using chi^2^ test. Correlations were evaluated using the Pearson’s test. A one-way ANOVA was applied for statistical analysis with the subsequent use of a post-hoc test, in order to statistically assess differences between groups; Tukey’s test was selected because of the uneven amount of data in individual groups. *p* < 0.05 was accepted as significant value.

## 5. Conclusions

In children with ISS, TSH and ghrelin secretion are closely related and a positive correlation between their concentrations was observed. It is interesting that the higher ghrelin and TSH concentrations, the lower the nocturnal GH and IGF-I secretion, however, without any impact on FT4 concentration.

It seems that ISS children in whom the FT4 concentration value is in the lowest tercile of the normal range, as well as ISS children with TSH level in the upper normal range (above the median value), do not require treatment with L-T4.

## Figures and Tables

**Table 1:** The auxologic and biochemical parameters in children with ISS, divided into two (2) groups distinguished by dependence on TSH concentration result: first one higher than or equal to the median value - 2.29 µIU/mL and second lower than 2.29 µIU/mL.

	Lower Normal TSH TSH < 2.29 µIU/mL	Higher Normal TSH TSH ≥ 2.29 µIU/mL	*p*
N (f/m)	43 (22/21)	42 (14/28)	
CA (years)	9.43 ± 2.98	8.85 ± 2.87	0.1147
Height SDS	−2.65 ± 0.92	−2.43 ± 0.69	0.2135
BMI SDS	−0.30 ± 1.09	−0.39 ± 0.71	0.6395
TSH (µIU/mL)	1.65 ± 0.51 *	3.22 ± 0.82 *	0.0000
FT4 (ng/mL)	1.30 ± 0.22	1.32 ± 0.16	0.6020
FT3 (pg/dL)	3.89 ± 0.56	4.16 ± 0.37	0.1993
FT4/FT3 ratio	0.34 ± 0.05	0.34 ± 0.04	0.7459
GHmax after clonidine (ng/mL)	19.56 ± 10.33	16.67 ± 7.91	0.1548
GHmax after glucagone (ng/mL)	12.15 ± 7.65	10.31 ± 7.09	0.2757
GHmax during sleep (ng/mL)	17.56 ± 10.31 *	12.46 ± 4.91 *	0.0048
IGF-I (ng/mL)	198.40 ± 107.33 *	148.13 ± 67.42 *	0.0144
IGFBP-3 (µg/dL)	4.59 ± 0.98	4.40 ± 1.58	0.5041
IGF-I/IGFBP-3 ratio	0.24 ± 0.11	0.20 ± 0.10	0.0944
Ghrelin (pg/mL)	1062.36 ± 443.43 *	1578.23 ± 807.37 *	0.0004
Leptin (ng/mL)	5.73 ± 8.09	3.50 ± 5.39	0.1583
Adiponectin (ng/mL)	18.10 ± 8.41	16.72 ± 7.27	0.4416
Resistin (ng/mL)	10.02 ± 3.89	10.27 ± 4.22	0.7856
AR index	0.76 ± 0.27	0.79 ± 0.25	0.5408
Triglycerides (mg/dL)	71.03 ± 26.54	64.57 ± 29.19	0.3729
Cholesterol (mg/dL)	159.73 ± 40.60	162.27 ± 29.72	0.7761
LDL-cholesterol (mg/dL)	92.30 ± 36.32	89.93 ± 25.52	0.7737
HDL-cholesterol (mg/dL)	57.83 ± 15.32	57.39 ± 16.87	0.9162
OGTT, n = 28 (f/m)	12 (6/6)	16 (8/8)	
Glucose 0′ (mg/dL)	82.32 ± 9.19	83.66 ± 9.55	0.5481
Glucose 60′ (mg/dL)	115.25 ± 36.51	137.31 ± 49.29	0.2039
Glucose 120′ (mg/dL)	91.00 ± 20.60 *	120.69 ± 28.73 *	0.0053
Insulin 0′ (µIU/mL)	5.72 ± 3.93 *	3.56 ± 1.90 *	0.0096
Insulin 60′ (µIU/mL)	33.34 ± 20.80	34.15 ± 16.24	0.9081
Insulin 120′ (µIU/mL)	20.15 ± 12.21 *	31.04 ± 15.12 *	0.0481
IRI HOMA	1.20 ± 0.84 *	0.76 ± 0.44 *	0.0158
IRI Belfiore	0.93 ± 0.37	1.05 ± 0.43	0.4570

CA—chronological age; BMI—body mass index; SDS—standard deviation score; IGF-I—insulin-like growth factor I; IGFBP-3—insulin-like growth factor binding protein-3; IRI—insulin resistance index; HOMA—homeostasis model assessment, AR index—adiponectin-resistin index; OGTT—oral glucose tolerance test; *—pairs of results marked with asterisks are characterized by a statistically significant difference; N—number of patients; *p*—level of statistical significance.

**Table 2:** The auxologic and biochemical parameters in children with ISS divided into two (2) groups distinguished by dependence on FT4 concentration: the first one—FT4 remaining in the lowest tercile of the reference range (i.e., below 1.1 ng/mL) and the second—FT4 equal to or being above the upper limit of lowest tercile - 1.1 ng/mL (i.e., FT4 within the middle or highest tercile of the reference range).

	Lower Normal FT4	Higher Normal FT4	*p*
	FT4 < 1.1 ng/mL	FT4 ≥ 1.1 ng/mL	
N	21	64	
CA (years)	9.80 ± 3.27	9.54 ± 2.94	0.7382
Height SDS	−2.74 ± 0.52	−2.49 ± 0.88	0.2364
BMI SDS	−0.44 ± 0.93	−0.31 ± 0.92	0.5978
TSH (µIU/mL)	2.16 ± 1.11	2.52 ± 1.02	0.1792
FT4 (ng/mL)	1.07 ± 0.15	1.3 ± 0.13	0.0000
FT3 (pg/dL)	3.50 ± 0.71	4.13 ± 0.36	0.0147
FT4/FT3 ratio	0.34 ± 0.06	0.34 ± 0.04	0.9778
GHmax after clonidine (ng/mL)	15.76 ± 7.63	18.84 ± 9.75	0.2007
GHmax after glucagone (ng/mL)	11.04 ± 6.54	11.34 ± 7.79	0.8744
GHmax during sleep (ng/mL)	15.97 ± 7.76	14.47 ± 8.46	0.4825
IGF-I (ng/mL)	191.05 ± 118.11	167.01 ± 83.72	0.3214
IGFBP-3 (µg/dL)	4.49 ± 0.90	4.50 ± 1.42	0.9817
IGF-I/IGFBP-3 ratio	0.23 ± 0.13	0.21 ± 0.10	0.4081
Ghrelin (pg/mL)	1224.93 ± 712.56	1357.95 ± 691.69	0.4581
Leptin (ng/mL)	5.12 ± 8.60	4.54 ± 6.55	0.7633
Adiponectin (ng/mL)	14.98±6.38	18.07 ± 8.19	0.1548
Resistin (ng/mL)	9.49 ± 4.13	10.27 ± 4.03	0.4841
AR index	0.80 ± 0.20	0.77 ± 0.28	0.6998
Triglycerides (mg/dL)	77.13 ± 36.27	64.41 ± 23.69	0.1186
Cholesterol (mg/dL)	172.33 ± 47.79	156.65 ± 29.93	0.1137
LDL-cholesterol (mg/dL)	103.13 ± 44.56	86.67 ± 23.73	0.0718
HDL-cholesterol (mg/dL)	56.25 ± 13.22	58.12 ± 16.98	0.6922
OGTT, n = 28 (f/m)	9 (5/7)	19 (9/7)	
Glucose 0′ (mg/dL)	84.56 ± 6.96	82.28 ± 10.01	0.3763
Glucose 60′ (mg/dL)	139.57 ± 48.49	123.95 ± 44.19	0.4358
Glucose 120′ (mg/dL)	110.29 ± 35.53	107.19 ± 27.81	0.8136
Insulin 0′ (µIU/mL)	4.30 ± 3.61	4.51 ± 2.90	0.8200
Insulin 60′ (µIU/mL)	34.85 ± 24.59	33.41 ± 16.20	0.8597
Insulin 120′ (µIU/mL)	26.15 ± 19.81	25.93±13.15	0.9741
IRI HOMA	0.93 ± 0.86	0.94 ± 0.58	0.9614
IRI Belfiore	0.83 ± 0.55	1.05 ± 0.33	0.2136

CA—chronological age; BMI—body mass index; SDS—standard deviation score; IGF-I—insulin like growth factor I; IGFBP-3—insulin-like growth factor binding protein 3; IRI—insulin resistance index; HOMA—homeostasis model assessment, AR index—adiponectin-resistin index; OGTT—oral glucose tolerance test; N—number of patients; *p*—level of statistical significance.
